# Quantum Cascade Laser-Based Photoacoustic Sensor for Trace Detection of Formaldehyde Gas

**DOI:** 10.3390/s90402697

**Published:** 2009-04-16

**Authors:** Angela Elia, Cinzia Di Franco, Vincenzo Spagnolo, Pietro Mario Lugarà, Gaetano Scamarcio

**Affiliations:** Dipartimento Interateneo di Fisica “M. Merlin”, Università and Politecnico of Bari, and Laboratorio Regionale CNR-INFM “LIT3”, Bari, I-70126, Italy; E-Mails: cinzia.difranco@fisica.uniba.it (C.-D.F.); spagnolo@fisica.uniba.it (V.S.); lugara@fisica.uniba.it (P.-M.L.); scamarcio@fisica.uniba.it (G.S.)

**Keywords:** Formaldehyde, Quantum cascade laser, Photoacoustic spectroscopy, Trace gas sensor

## Abstract

We report on the development of a photoacoustic sensor for the detection of formaldehyde (CH_2_O) using a thermoelectrically cooled distributed-feedback quantum cascade laser operating in pulsed mode at 5.6 μm. A resonant photoacoustic cell, equipped with four electret microphones, is excited in its first longitudinal mode at 1,380 Hz. The absorption line at 1,778.9 cm^−1^ is selected for CH_2_O detection. A detection limit of 150 parts per billion in volume in nitrogen is achieved using a 10 seconds time constant and 4 mW laser power. Measurements in ambient air will require water vapour filters.

## 1. Introduction

Formaldehyde (CH_2_O) is a carcinogenic pollutant emitted as an intermediate product in the oxidation of most biogenic and anthropogenic hydrocarbons. It is also known as a primary emission product of incomplete hydrocarbon combustion [1]. This makes formaldehyde an ubiquitous component of both remote and polluted urban atmospheric environments. The CH_2_O concentrations in polluted urban environments are in the order of 10 – 20 ppbv (parts per billion in volume), whereas in non-urban locations concentrations from 0.01 to 10 ppbv have been observed [2–5]. Moreover, formaldehyde is a chemical widely used in the manufacture of building materials and many household products like foams, consumer paints and polymer products. Outgassing of formaldehyde from these materials may lead to elevated CH_2_O concentration levels in indoor air. Even at low concentrations, formaldehyde can lead to health risks and may be associated with various diseases, such as bronchial asthma, atopic dermatitis and “sick building” syndrome. At concentrations of 100 – 500 ppbv irritation of eyes, nose, and throat has been reported. At higher concentrations CH_2_O leads to headaches and dizziness, and at 100 ppmv (parts per million in volume) exposure can be fatal [6]. Thus, strict regulation and controls on CH_2_O emissions are required. As an example, an upper limit of 0.75 ppmv for long-term exposure (8 h time-weighted average) and 2 ppmv for short-term exposure (15 min) has been imposed by the US Occupational Safety and Health Administration to protect workers from exposure to formaldehyde [7].

A number of different methods for monitoring the formaldehyde level in both the environment and industrial exhausts have been developed and validated [8–10], e.g. gas absorption on solid or liquid matrix, extraction by filtration and/or diffusion, specific derivatization reactions to form a detectable compound and finally the detection by chromatographic techniques coupled with mass spectrometry.

The detection limits of the wet chemical techniques are in the ppbv range; however they suffer from interference from the environmental conditions (temperature, humidity), are expensive and have to be performed by highly specialized personnel. These methods also exhibit slow response times, typically on the order of minutes, related with the chromatographic separation and this prevents application requiring real–time and continuous gas monitoring.

To overcome these limitations several laser-based spectroscopic systems have been developed. Among them, direct absorption and cavity enhancement spectroscopies (i.e. cavity ring down spectroscopy) take advantage of long optical path in multi-pass cell and high finesse optical cavities, respectively. These techniques allow high sensitivities (up to sub-ppbv), however they need sophisticated and/or cumbersome equipments, not suitable in applications which require compact and transportable sensors [11,12]. For example, multi-pass absorption spectroscopy requires high volume multi-pass cell and sensitive IR detectors like thermoelectrically cooled or room-temperature photoconductive detectors or even liquid nitrogen cooled mercury cadmium telluride detectors. Instead, the major drawbacks of the cavity ring down spectroscopy are the requirement for high-reflectivity mirrors and high-quality laser beam.

On the other hand, photoacoustic spectroscopy (PAS) has the potentiality to result in simple, robust, cheaper and easy to maintain designs, less sensitive to the problems of interference fringes and optical misalignments, giving PAS a competitive advantage over other sensitive techniques and the possibility to obtain a man-portable sensor.

Moreover, while the sensitivity of direct absorption technique is independent from laser optical power, PA spectroscopy benefits from the use of high intensity sources to reach lower detection limits, since its sensitivity scales linearly with the laser power.

In the last few years, efficient quantum cascade laser (QCL) sources, emitting in the mid-IR molecular fingerprint region, have become available. These lasers work at room temperature with emitted power up to several Watts [13] and thus represent ideal sources for PA gas sensing; detection limits of few ppbv [14–16] have been already demonstrated.

In this work we report the development and calibration of a PA trace gas sensor for the monitoring of formaldehyde with a detection limit of 150 ppbv, based on a resonant cell and a commercial QC laser source emitting at 1,778.9 cm^−1^. The sensor easily meets the international environmental regulations in terms of minimum detectable CH_2_O concentration.

## 2. Experimental

**Figure 1.**
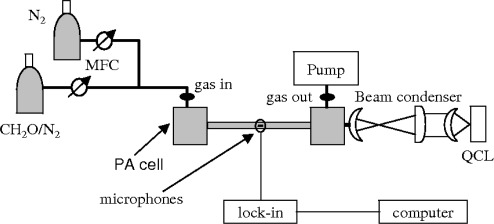
Schematic diagram of the photoacoustic sensor. The sensor is about 0.5 m long, 0.2 m high and 0.2 m wide.

The experimental set-up is depicted in Figure 1. The photoacoustic spectrometer consists of a resonant cell and a commercial distributed feedback QCL, supplied by Alpes Laser (Neuchatel, Switzerland), with dedicated drive electronics for pulsed operation. In order to maximize the laser power and limiting chirping effects, we worked with pulse duration of 42 ns and a duty cycle of 1.4%. A Peltier cooled aluminium housing held the laser device at a constant temperature. The laser radiation was collected with an AR coated ZnSe lens (2.54 cm focal length, f/1) and collimated by a beam condenser (0.2X) to avoid reflections on the cell walls. The laser beam intensity was electronically modulated at the first longitudinal resonance frequency of the PA cell.

The resonant cell consists of a cylindrical stainless steel resonator of 120 mm length and 4 mm radius, with two 60 mm (λ/4) long buffer volumes connected to its endings, in order to reduce by destructive interference the background signal due to the heating of the two ZnSe windows sealing the cell. The CH_2_O molecule is a notoriously adhesive molecule; thus a major problem is the accurate measurement of ultrasmall concentrations. To reduce the influence of CH_2_O adsorption at the surfaces, we realize a PA cell similar to that used in ref. 16, but with the inner walls of the cell gold coated. We also optimized the buffer dimensions and inlet-outlet gas system, in order to less influence the cell acoustic modes.

The resonator was designed to be excited in its first longitudinal mode at 1380 Hz; it was equipped with four electret microphones (Knowles EK 3024), with a reported sensitivity of S_m_ = 20 mV/Pa, placed on the antinode of the acoustic mode, to increase the signal-to-noise ratio (SNR). The electrical signal, fed by the microphones, was pre-amplified and then measured by a digital lock-in amplifier (EG&G Instruments), with an integration time constant τ_int_ = 10 s.

A certified 99.8-ppmv CH_2_O in N_2_ mixture was used to obtain known concentrations of the investigated gas in the 0.25 – 10 ppmv range via two mass flow controllers (MFC). We used a chemical trap (Entegris mod. 35kf) to further reduce the water vapour concentration in the certified mixture down to 0.1 ppb. The pressure in the PA cell was kept at 1 atm. The purging of the system was accomplished by a small diaphragm vacuum pump.

## 3. Results and Discussion

### 3.1. Analysis of Spectroscopic Data

**Figure 2.**
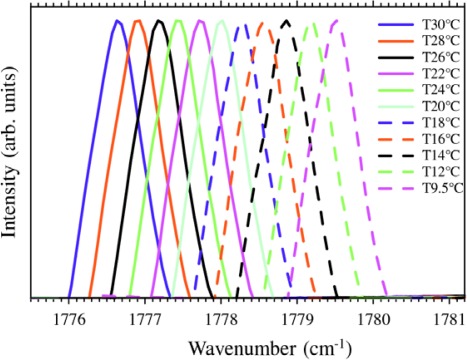
Laser spectra measured at different temperatures, recorded by Fourier-Transform infrared spectrometer with a resolution of 0.125 cm^−1^. Pulse width of 42 ns and 1.4% duty cycle were used. The related device temperatures are shown in the legend, starting from the rightmost spectrum. The temperature-tuning coefficient is −0.140 cm^−1^/°C.

The QCL used in this paper works in single mode emission at a wavelength around 5.6 μm, where the formaldehyde C=O stretching mode (ν_2_ fundamental band) is located [17]. For highly sensitive spectroscopic detection of CH_2_O, intense absorption lines and free from cross-interferences of other gases have to be selected. Wavelength mappings of the PA spectra require accurate knowledge of the dependence of the QCL emission wavelength on the temperature. The shift of the laser wavelength versus the device temperature was investigated in the range 10 – 30 °C. The laser spectra were analyzed with a Fourier-Transform infrared spectrometer (Nicolet Instruments Inc.) with a resolution of 0.125 cm^−1^. Figure 2 shows a selection of the spectra measured at different temperatures. The single lasing mode shifts linearly from 1,779.5 cm^−1^ at 9.5 °C to 1,776.6 cm^−1^ at 30 °C with a tuning coefficient of −0.140 cm^−1^/°C. The lasing mode suffers from an increase in linewidth as a result of thermal chirping which is typical of pulsed QCLs.

**Figure 3.**
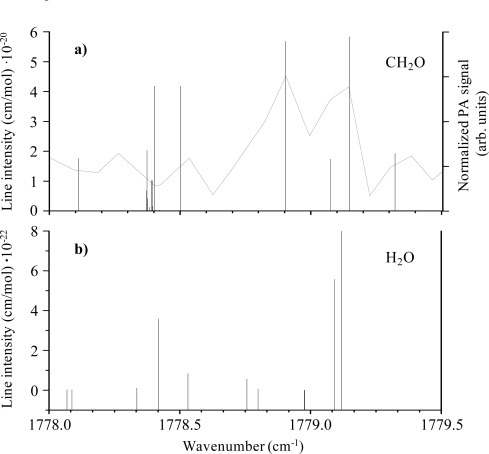
a) Normalized PA spectrum of 10-ppmv formaldehyde (solid curve) recorded 1,778 – 1,779.5 cm^−1^ by varying the temperature of the laser in steps of 0.5 degrees. The individual vertical lines mark the CH_2_O absorption lines reported in reference 18. b) HITRAN H_2_O spectrum.

Since strong H_2_O absorptions fall in the spectral region of laser tunability, we performed an analysis of reference spectra of CH_2_O (panel a of Figure 3) and H_2_O (panel b of Figure 3), obtained from [18] and the HITRAN database [19], respectively, in order to evaluate their possible interferences. In order to choose a CH_2_O absorption line free from interferences and suitable for high PA response, we also performed a study of formaldehyde PA spectrum of CH_2_O in the spectral region of laser tunability.

The PA 10-ppmv formaldehyde spectrum was recorded in the spectral range 1,778 – 1,779.5 cm^−1^ by varying the temperature of the laser in steps of 0.5 degrees (panel a of Figure 3). The spectral linewidth results from the convolution of the CH_2_O absorption linewidth the laser linewidth. The intensity, related to CH_2_O line strengths, has been normalized to QCL optical power. The measured CH_2_O spectrum shows a good agreement with the line positions reported by Perrin *et al.* [18]. The normalized PA signal at the 1,778.9 cm^−1^ line is nearly identical to than around 1,779.1 cm^−1^, according to the correlated CH_2_O line strengths. We select the CH_2_O peak at 1,778.9 cm^−1^ for PA detection experiments due to its smaller overlap with water lines. According to CH_2_O Perrin’s data a 5.68 × 10^−20^ cm/molecule intense line is associated. The lasing emission has been fixed over this line by setting the temperature of our QC laser at T = 13.7 °C.

### 3.2. Calibration of PA sensor

Calibration measurements were carried out by further diluting the CH_2_O mixture with pure nitrogen. The concentration was reduced from 5 to 0.25 ppmv and the magnitude of the PA signal was measured by the lock-in amplifier with a time constant τ_int_ = 10 s. The background signal, mainly due to the periodical heating of the PA cell windows and walls, was measured by filling the cell with pure nitrogen and results essentially constant during the calibration measurements and coherent with the PA signal. The electronic noise, due to external acoustic and electromagnetic sources is mainly uncorrelated with the modulation frequency and was detected with the laser beam off.

**Figure 4.**
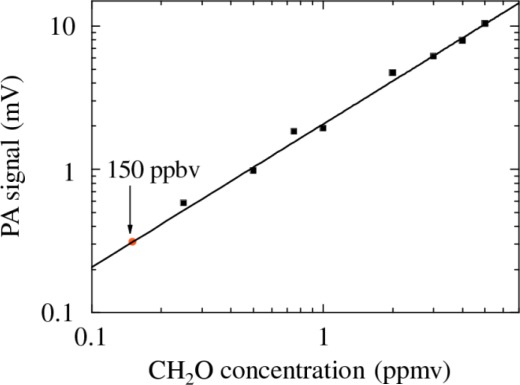
PA signal versus CH_2_O concentration. The symbols represent experimental data, solid line is a linear fit. The error bars corresponding to the uncertainty on PA signal have the same dimensions of the symbols. The vertical arrow marks the noise equivalent (signal-to-noise ratio of 1) minimum detection limit of 150 ppbv.

Figure 4 shows the PA signal measured as a function of CH_2_O concentration down to 250 ppbv and corrected for the in-phase component of the background. These measurements were performed after an accurate heating and purging of the cell to avoid contributions from adsorbed-desorbed molecules. The solid line is a linear fit of the data; a strong linear relationship (r^2^ = 0.999) between the PA signal and the CH_2_O concentration results.

We evaluate the detection limit of our sensor and the interferences from the presence of water vapour. H_2_O in concentration of 1,000 ppmv produced a PA signal comparable to 10 ppmv of CH_2_O. Thus, concentration measurements in ambient or open air samples should require water vapour filters, such as a compact thermoelectric cooled traps, which enable to remove a large fraction of the water vapour (down to few hundreds of ppmv), combined with a chemical filter and/or a differential PA technique. The use of the above water removal systems give no losses in the analyte compounds and measurements of ambient air, with different CH_2_O concentrations, filtered by the above water removal systems show that a negligible water vapour contribution to the overall PA signal is obtained. The detection limit *C* for CH_2_O diluted in N_2_ was calculated considering a *SNR* = 1 from the following equation:

$$C=\frac{\mathrm{SNR}\sigma }{a}$$

where *σ* is the standard deviation of the linear fit and *a* the slope of the calibration curve (calibration factor). An extrapolated detection limit of 150 ppbv was found.

Since the PA signal is proportional to the optical excitation power, sensor performance can be more efficiently described by the laser-independent value given by the minimum detectable absorption coefficient, normalized to power and detection bandwidth. In fact, the minimum optical absorption coefficient detectable with a PAS based sensor is determined by the condition *S* = *N*, where *S* is a PA signal and *N* is the noise level. Usually the noise power density can be considered constant within the detector bandwidth Δ*f*. In this case the average noise magnitude *N* ≈ (Δ*f*)^1/2^.

The normalized noise equivalent absorption coefficient is given by:

$$D=\frac{\alpha_{\text{min}}P_{0}}{\sqrt{\Delta f}}$$

where *α_min_* is the minimum detectable absorption coefficient at a SNR = 1, *P_0_* the average laser optical power and Δ*f* the equivalent noise detection bandwidth.

In this case, considering *α_min_* = 6.6 × 10^−7^ cm^−1^, *P_0_* = 4 mW and Δ*f* = 0.017 Hz (*τ_int_* = 10 s, 12-dB/octave digital filter) we obtained a detection sensitivity of D = 2.0·10^−8^ W·cm^−1^Hz^−1/2^. Table 1 summarises the sensor performances. To further characterize our sensor we performed a set of measurements for nitric oxide (NO) gas traces. The obtained results are also shown in Table 1. We obtained better results with respect to ref. 16, mostly due to improvements in the PA cell and reduction of electromagnetic noise sources. The lower *α*_min_ and consequently D obtained for NO with respect to CH_2_O is due to the lower standard deviation of the linear fit, thanks to the higher signal-to-noise ratio measured for NO.

Our CH_2_O sensor results compact (∼ 0.05 m^3^ including a thermoelectric cooled/chemical trap water removal system) and exhibits parameters comparable with that obtained by Horstjann *et al.* [20] via a quartz-enhanced PA fork system coupled with a liquid nitrogen cooled (78 K) inter-cascade laser (ICL) (D = 2.2 × 10^−8^ Wcm^−1^Hz^−1/2^ for SNR = 1) and lower than that reported by Angelmahr *et al.* [21] which developed a CH_2_O PA sensor based on a differential resonant cell (D = 6.2 × 10^−9^ Wcm^−1^Hz^−1/2^ for SNR = 1) and a grazing-incidence optical parametric oscillator (GIOPO) laser source. However, both these sensors are based on not-commercial laser sources, which required cryogenic cooling (as for the ICL) or rather complicated non-linear optical setup (GIOPO).

## 4. Conclusions

A trace level formaldehyde sensor, based on a commercial QCL working at RT and with a PA resonant cell, has been developed and calibrated. The laser wavelength has been carefully selected in order to achieve strong CH_2_O absorption, while keeping the possible interferences from water vapour as low as possible. We report a detection limit of 150 ppbv in nitrogen, which comfortably meets current industry and environmental regulations. CH_2_O measurements in ambient air require water vapour filters due to interferences with H_2_O absorption lines.

Future developments to improve the obtained sensor performances will be the implementation of: i) a QC laser operating at room temperature in continuous mode, in order to eliminate the thermal chirping of pulsed operation, increase the sensor selectivity and significantly improve the signal-to-noise ratio thanks to the higher optical power; ii) optical microphone, based on the detection of the vibration of a membrane using light and characterized by high Sensibility (5 V/Pa), to improve the SNR; iii) fiber coupling with collimating system between the QC laser and the PA cell, in order to take rid of any optical alignment issue and thus realizing a more compact, robust and portable PA sensor.

## Figures and Tables

**Table 1.** Summary of the PA sensor performances.

**PA sensor performance**		
	CH_2_O	NO
P_0_ (mW)	4	2
S_m_ (mV/Pa)	20	20
τ_int_ (s)	10	10
α_min_ (cm^−1^)	6.6 × 10^−7^	1.3 × 10^−7^
D (W·cm^−1^Hz^−1/2^)	2.0 × 10^−8^	2.0 × 10^−9^
Detection limit (ppbv)	150	150
